# Detection of Multiple Human Papillomavirus Genotypes in Anal Carcinoma

**DOI:** 10.1186/1750-9378-5-17

**Published:** 2010-10-12

**Authors:** Sonia Ramamoorthy, Yu-Tsueng Liu, Linda Luo, Katsumi Miyai, Qing Lu, John M Carethers

**Affiliations:** 1 Departments of Surgery, University of California, 9500 Gilman Drive, San Diego, California, 92093-0068, USA; 2 Departments of Medicine, University of California, 9500 Gilman Drive, San Diego, California, 92093-0068, USA; 3 Departments of Pathology, University of California, 9500 Gilman Drive, San Diego, California, 92093-0068, USA; 4 Departments of Rebecca and John Moores Comprehensive Cancer Center, University of California, 9500 Gilman Drive, San Diego, California, 92093-0068, USA; 5 Department of Internal Medicine, University of Michigan Medical School, 1500 E. Medical Center Drive, Ann Arbor, Michigan, 48103-5368, USA

## Abstract

Infection with human papillomavirus (HPV) is a major risk factor for development of anal squamous cell carcinoma. Despite over 100 genotypes of the virus, HPV 16 and 18 are considered pathogenic as they are seen in the majority of cervical and anal cancers. We have employed a custom microarray to examine DNA for several HPV genotypes. We aimed to determine the accuracy of our microarray in anal cancer DNA for HPV genotypes compared to the DNA sequencing gold standard.

**Methods:**

We utilized a sensitive microarray platform to classify 37 types of mucosal HPVs including 14 known high-risk and 23 low-risk types based on cervical cancer data. We utilized DNA from pathologically confirmed cases of anal squamous cell carcinoma. All samples underwent microarray HPV genotyping and PCR analysis.

**Results:**

HPV was detected in 18/20 (90%) anal cancers. HPV genotypes 16 and 18 were present in the majority of specimens, with HPV 16 being the most common. Eighty percent of anal cancers had at least two HPV types. Ten percent of cases (2/20) tested negative using our microarray; DNA sequencing confirmed the lack of presence of HPV DNA in these samples.

**Conclusions:**

Microarray technology is an accurate way to screen for various genotypes of HPV in anal cancer, with 100% correlation with genomic DNA detection of HPV. The majority of anal cancers in our study associated with pathogenic HPV 16 and/or 18. Other HPV genotypes are present simultaneously with HPV 16 and 18, and might contribute to its pathogenesis.

## Introduction

Squamous cell cancer of the anus (anal cancer) is increasing in frequency in the general population in the United States, Europe and South America [1]. There were an estimated 4000 new cases in 2003 in the United States [2], and that number was estimated to be 5290 for 2009 [3]. Despite the increasing numbers of patients with anal cancer, little has changed in the paradigm for the treatment and outcomes from this disease. Anal cancer was initially thought to develop from conditions of chronic inflammation such as perianal Crohn's disease; however genital viral infection with HPV with or without a concomitant immunocompromised condition has been shown to be the major risk factor for the development of anal dysplasia, a precursor lesion to squamous cell cancer of the anus. There are likely over 100 genotypes of the virus, however types HPV 16 and 18 are considered "high risk" as they are seen in the majority of cervical and anal cancer specimens [4]. Little data exists confirming the presence of these types or other genotypes in squamous cell cancer of the anus. With cervical cancers, line probe assays and reverse line blot (RLB) assays have been utilized, with RLB assays better at detecting multiple HPV infections, but none of these tests are universally agreed upon for detection purposes [5]. To date, most studies in anal cancer have used DNA hybridization, PCR techniques and DNA sequencing to identify and confirm the presence or absence of HPV 16, 18 [6,7]. While most anogenital cancers are believed to be associated with HPV 16 and 18, the additive presence of other "high risk" types is unknown. We have developed and utilized a microarray platform to examine DNA for HPV genotyping in anal carcinomas. In this study, we aimed to determine the accuracy of our microarray technology to examine anal cancer DNA for HPV types using DNA sequencing techniques as the gold standard.

## Methods

### DNA Extraction

Under IRB approval, DNA was extracted from pathologically confirmed cases of anal cancer from two local hospitals: UC San Diego Medical Center and the Veteran's Administration Hospital in San Diego, California. A total of 20 patient samples were collected from these two cancer registries for use in this study. The average age of the patients was 51.5 (range 39-77) and 85% of patients were males. Staging was performed by computed tomography, and listed by AJCC classification in Table 1. All samples were taken before treatment with chemoradiation; in some cases the lesions were treated with surgery alone. Of the 20 cases, 45% (N = 9) were known to be HIV positive. All samples were histologically confirmed by a single pathologist prior to experiments (KM).

**Table 1:** HPV infection and genotype among our cohort of human anal cancers.

CASE	HPV STATUS BY PCR	HPV GENOTYPE BY MICROARRAY	AJCC Stage*
1	Positive	11, 16, 43	T2N2M0 = 3B
2	Positive	18	T3N1M0 = 3A
3	Positive	16	T1N0M0 = 1
4	Positive	16, 18	T2N0M0 = 1
5	Positive	16,18	T3N0M0 = 2
6	Positive	16, 18	T2N1M0 = 3A
7	Positive	16, 18	T3N0M0 = 2
8	Positive	18, 33	T2N0M0 = 1
9	Positive	16, 18	T2N0M0 = 1
10	Positive	16, 18	T1N0M0 = 1
11	Positive	6, 16	T2N1M0 = 3
12	Positive	16, 33	T2N0M0 = 1
13	Positive	11, 16	T3N1M1 = 4
14	Positive	16, 18	T3N0M0 = 2
15	Positive	16, 18	T2N1M1 = 4
16	Positive	16, 18	T2N0M0 = 2
17	Positive	16, 18	T1N0M0 = 1
18	Positive	16, 18	T2N2M0 = 3B
19	Negative	Negative	T2N0M0 = 1
20	Negative	Negative	T3N0M0 = 2

* Reference American Joint Committee on Cancer: AJCC Cancer Staging Manual. 6th ed. New York, NY: Springer, 2002, pp 125-130.

DNA tissue was extracted after manual or laser capture microscopy microdissection of pathologist-marked cancer areas from histological-confirmed slides of squamous cell cancer of the anus. This tissue was then deparaffinized in xylene, purified with absolute alcohol, and centrifuged at 15,000 rpm. The pellets were dried in a DNA SpeedVac (Eppendorf Vacufuge Concentrator), re-suspended in 100 μl Tris EDTA buffer, and incubated with 200 ug/ml proteinase K (Sigma Cat#P2308) at 55°C for 3 hours. The genomic DNA was purified with phenol/chloroform/isoamyl alcohol (25:24:1, Sigma cat, P-3803) and washed with alcohol. The resulting solute was re-suspended in TE buffer. Purified DNA was then subject to amplification for microarray analysis.

DNA was also extracted from cell lines in which the HPV status was known. SiHa (HPV16, 1-2 copies/cell), HeLa (HPV-18, 10-50 copies/cell), CaSki (HPV-16, 60-600/cell) and ME180 (HPV-68) were obtained from ATCC and grown in their ATCC-recommended media at 37°C in 5% CO_2_.

### HPV DNA Amplification

We used a modification of the standard GP5+/GP6+ protocol [8]. Modified primers, termed B-GP5+ and B-GP6+, containing a 5' extension of 17mer primer B sequence (GTTTCCCAGTCACGATC) to the original GP5+ and GP6+ primers, were used for the initial PCR amplification from samples. The initial PCR product was further amplified and labeled with amino-allyl dUTP by using the B primer for the 2^nd ^PCR. Subsequently, the labeled product was then coupled with Cy-3 NHS ester for array hybridization. This approach demonstrates the usefulness of this method for detection of the amplified viral amplicons when multiple viral types or genotypes might be present [9,10].

### HPV Genotyping Chip

**Figure 1:**
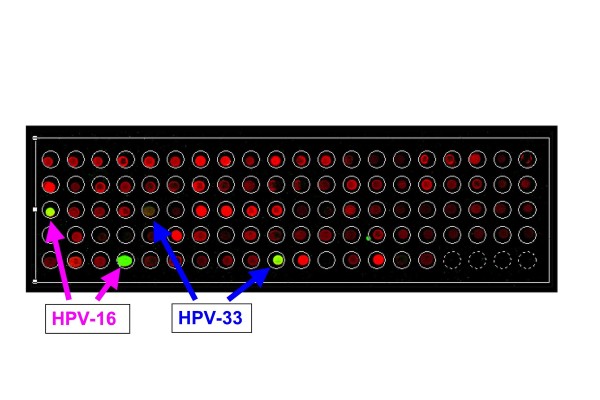
**DNA HPV Microarray of Human Anal Cancer**. As explained in the Methods section, the yellow-green spots on the microarray indicate positive hybridization for HPV. This human anal cancer sample is positive for HPV-16 and HPV-33. Each HPV genotype is represented by two single-stranded oligonucleotide probes (i.e. forward and reverse complementary orientations).

A microarray platform was built to type 37 types of mucosal HPVs (see Figure 1) that include 14 presumed high-risk types: 16, 18, 31, 33, 35, 39, 45, 51, 52, 56, 58, 59, 66 and 68; and 23 presumed low-risk types: 6, 11, 26, 34, 40, 42, 43, 44, 53, 54, 55, 57, 61, 70, 71, 72, 73, 81, 82/MM4, 82/IS39, 83, 84 and CP6108. The probes were all 30-mer in length including forward and reverse orientations and were located in a 150 bp L1 fragment that is bordered by primers GP5+ and GP6+ as previously described [8]. Each HPV type is represented by a pair of probes (for example, two HPV16 spots indicated in Figure 1), which correspond to one set of oligonucleotide sequences complementary to each other. The location of each probe is identified by the scanner software using a premade grid file without special pattern arrangement. Prior to printing, every HPV-specific oligonucleotide probe was mixed with a Spike-70 oligonucleotide probe at the ratio of 50:1 for a given spot on the glass slide [11]. Probe-70 is labeled with Cy-5 dye (red in Figure 1) and is the reverse complement sequence to Spike-70. Probe-70 is mixed with HPV DNA (labeled with Cy3, green in Figure 1) amplified from samples. Since all spots contain probes for Spike-70, all spots show up in the red channel when scanned. However, if the spot contains probes for an HPV type in the sample, then the green signal in the HPV labeled DNA merges with the red signal from the Spike-70:Probe-70 hybridization to yield a yellowish spot dependent on the ratio of green to red. Each probe was spotted four times on the chip (Figure 1). The microarray fabrication procedure has been described previously [9,11].

## Results

### HPV genotyping and detection by microarray

We used SiHa (a known HPV 16 positive cervical cancer cell line) genomic DNA for analyzing the limit of detection to mimic the complexity of HPV16 in the presence of human genomic DNA. SiHa cells contain a single HPV-16 integrated into chromosome 13q [12]. The human genome is about 3.3 billions base pairs, therefore, each ng of genomic DNA contains about 3 × 10^5 ^molecules of human genome. A total of 100 ng SiHa genomic DNA was sequentially diluted in water, amplified, labeled and hybridized to the HPV typing chip. SiHa (1-2 integrated HPV16/cell) genomic DNA was applied for titration experiment. The sensitivity with the HPVGP56 array is about 1-2 copies of HPV16 in the background of complex human genomic DNA. (The detection limit was about 6.4 pg of genomic DNA, which is equivalent to 2 copies of haploid human genome, 3.6 pg/haploid genome). We were able to unambiguously identify features representing HPV16 when only 1 or 2 copies of genome were present in the sample.

To test the specificity of the array, 100 ng each of genomic DNA from SiHa (HPV16, 1-2 copies/cell), HeLa (HPV-18, 10-50 copies/cell), Caski (HPV-16, 60-600/cell) and ME180 (HPV-68) were used for amplification, labeling and chip hybridization [12,13]. The microarray results accurately identified each of the respective HPV types from each cell line. One of the array typing results from clinical samples is shown in Figure 1, with this particular tumor sample was positive for HPV 16 and HPV 33.

### HPV typing of anal cancer

**Figure 2:**
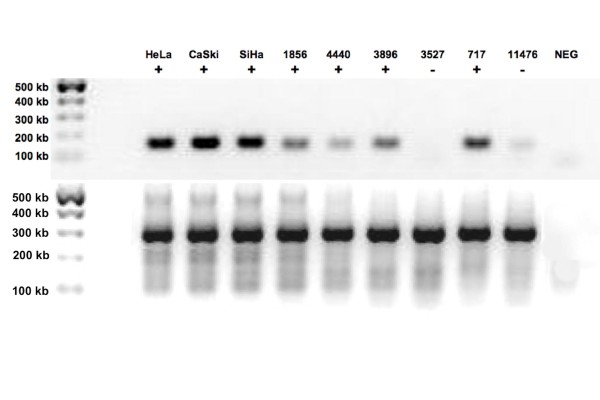
**GP5+/GP6+ PCR for HPV**. Human anal squamous genomic DNA samples were tested for the presence of HPV DNA. Hela, CaSki, and SiHa cells are all HPV positive cervical cancer controls. The "+" indicates positive samples, and the "-" indicates negative samples for HPV amplification in the upper panel. The lower panel demonstrates actin as an amplification control of the same samples. The leftmost lane contains a 100 base pair ladder marker. NEG demarcates a negative control without sample DNA.

After extracting the DNA for anal cancer specimens and hybridizing to the microarray, HPV was detected in 18/20 (90%) cases. All the samples were separately subject to PCR amplification and DNA sequencing analysis to confirm the presence or absence of HPV DNA in the cancer specimens and test the accuracy of the microarray platform. Genomic DNA from the anal cancer samples was used for GP5+/6+ primer PCR [14], a conserved region within HPV, both positive controls for HPV cancer cell lines (Hela, CaSki, SiHa) and negative controls were used to validate results (Figure 2).

DNA sequencing confirmed a 100% correlation between the HPV microarray and DNA sequencing analysis (Figure 2 and Table 1). Of the two cases that had no HPV detected by microarray, lack of HPV DNA was further confirmed by DNA sequencing analysis, suggesting 100% specificity (Table 1). Of the cases that were positive for HPV, 100% were initially detected by HPV microarray, with confirmation via DNA amplification and DNA sequencing.

We identified that anal cancers often contain more than one HPV genotype. Types 16 and 18 were present in the majority of our specimens, accounting for approximately 80% of the HPV detected, with HPV 16 being the most common (Table 1). Only 10% of cases demonstrated a single genotype infection with either HPV 16 or 18 alone, whereas more than 50% of our patients were infected with both HPV 16 and 18 and/or another high risk type such as HPV 33 (Table 1). Overall, approximately 80% of the anal cancers demonstrated more than one HPV genotype. All extracted tissue specimens underwent DNA sequencing to confirm the microarray findings. We did not find a correlation between the AJCC stage and HPV genotype.

## Discussion

A major risk factor for squamous cell cancer of the anus is HPV infection. HPV's E6 and E7 oncoproteins can inactivate crucial cell cycle regulators such as p53 and pRb, telomere maintenance, apoptosis and induce chromosome instability [2,6]. Anal cancer is commonly associated with HPV genotypes 16 and 18. Although as many as 100 different genotypes may exist, there has been no previous evaluation of multiple types that could be present in anal cancer. We developed a relatively rapid way to evaluate multiple HPV genotypes at once within anal cancer and studied whether this correlated with the gold standard of individual HPV DNA sequencing. We found 100% agreement between our microarray method when compared to DNA sequencing. Utilizing microarray technology to subtype the presence of HPV in squamous cell cancer of the anus, we detected a high rate of HPV infection within anal cancer, with 90% in our cohort of patients. This is comparable to previous reports demonstrating HPV infection rates from 75-80% [15,16].

The frequency of HPV detection in anal cancer underscores its dominance as a risk factor for this disease. We detected HPV types 16 and 18 in 70% of anal cancers, consistent with these genotypes associated with anal cancer pathogenesis. A recent meta-analysis suggests that HPV16 is found more frequently (75%) and HPV18 less frequently (10%) in anal carcinomas than in cervical carcinomas [17]. However, only 10% of our cohort of anal cancers had HPV type 16 or 18 alone. Indeed, HPV types 16 and 18 together infected more than 50% of anal cancers, which may or may not have implications in its pathogenesis. Furthermore, approximately 80% of anal cancers demonstrated more than one HPV genotype, including some infected with another high-risk type such as HPV 33. The implication of this information is unknown but the presence of multiple types may affect disease progression, immunity and treatment response. In the era of vaccines, the information about multiple HPV genotype infections may become increasingly important.

The analysis of HPV infection can be hindered by the lack of consistency in collection and detection methods, this is in part due to sample adequacy [18]. The microarray technology used in this study detected the presence of HPV in anal cancer DNA that was extracted from formalin-fixed tissue taken from biopsy specimens. This suggests that this method can be quite effective with only a minimal sized sample and can detect multiple HPV genotypes simultaneously. Previous studies have demonstrated the presence of HPV genotypes in anal cancer through PCR-based genotyping and commercially available gene chip analysis, however this can be expensive and time consuming for use in the clinical setting [19-21]. We believe that if uniformly applied in pathological laboratories, a microarray approach would be less expensive plus have the added benefit of detecting multiple genotypes of HPV. The application of a microarray platform for HPV typing could have significant implications for the prevention of HPV-related disease. The ability to identify and risk stratify those patients with HPV 16 and HPV 18 and possibly other genotypes may in the future, provide an opportunity for surveillance and early intervention. It is not yet clear if there is a genotype-stage correlation.

In summary, microarray technology is a novel and accurate way to screen for the various genotypes of HPV in anal cancer simultaneously, with a 100% correlation with genomic DNA detection of HPV in this cohort. The majority of anal cancers in our study is associated with HPV subtypes 16 and/or 18 and like cervical cancer, are the major genotypes associated with the cancer. Other HPV genotypes can be present in anal cancer and often occur simultaneously with genotypes 16 and 18, and might contribute to the progression of pathogenesis.

## Competing interests

The authors declare that they have no competing interests.

## Authors' contributions

Study concept and design: SR, Y-TL, JMC; acquisition of data: SR, Y-TL, LL, QL; analysis and interpretation of data: SR, Y-TL, LL, KM, QL, JMC; drafting of the manuscript: SR, JMC; critical revision of the manuscript for important intellectual content: SR, Y-TL, JMC; obtained funding: SR, Y-TL, JMC. All authors have read and approved the final manuscript.
